# Myocardial Extracellular Volume Fraction and T1 Mapping by Cardiac Magnetic Resonance Compared Between Patients With and Without Type 2 Diabetes, and the Effect of ECV and T2D on Cardiovascular Outcomes

**DOI:** 10.3389/fcvm.2021.771363

**Published:** 2021-12-07

**Authors:** Issarayus Laohabut, Thammarak Songsangjinda, Yodying Kaolawanich, Ahthit Yindeengam, Rungroj Krittayaphong

**Affiliations:** ^1^Division of Cardiology, Department of Medicine, Faculty of Medicine Siriraj Hospital, Mahidol University, Bangkok, Thailand; ^2^Faculty of Medicine Siriraj Hospital, Her Majesty Cardiac Center, Mahidol University, Bangkok, Thailand

**Keywords:** myocardial extracellular volume fraction, type 2 diabetes, cardiovascular outcomes, cardiac magnetic resonance, T1 mapping

## Abstract

**Background:** To investigate the difference in myocardial extracellular volume fraction (ECV) by cardiac magnetic resonance (CMR) T1 mapping between patients with and without type 2 diabetes (T2D), and the effect of ECV and T2D on cardiovascular (CV) outcomes.

**Methods:** All patients aged > 18 years with known or suspected coronary artery disease who underwent CMR for assessment of myocardial ischemia or myocardial viability at the Department of Cardiology of the Faculty of Medicine Siriraj Hospital, Mahidol University, Bangkok, Thailand from September 2017 to December 2018 were screened for inclusion eligibility. Left ventricular ejection fraction (LVEF), late gadolinium enhancement, and T1 mapping were performed. ECV values were derived from myocardial native T1 and contrast-enhanced T1 values that were obtained using modified Look-Locker inversion recovery at the septum of the mid-cavity short-axis map. Demographic data, clinical characteristics, and CV outcomes were collected by retrospective chart review. Composite CV outcomes included CV death, acute coronary syndrome, heart failure hospitalization, or ventricular tachycardia (VT)/ventricular fibrillation.

**Results:** A total of 739 subjects (mean age: 69.5 ± 14.0 years, 49.3% men) were included. Of those, 188 subjects had T2D (25.4%). ECV was significantly higher in T2D than in non-T2D (30.0 ± 5.9% vs. 28.8 ± 4.7%, *p* = 0.004). During the mean follow-up duration of 26.2 ± 8.5 months, 43 patients (5.8%) had a clinical composite outcome, as follows: three CV death (0.4%), seven acute coronary syndrome (0.9%), 33 heart failure hospitalization (4.5%), and one VT (0.1%). T2D, low LVEF, and high ECV were all identified as independent predictors of CV events. Patients with T2D and high ECV had the highest risk of CV events.

**Conclusion:** Among patients with known or suspected coronary artery disease, patients with T2D had a higher ECV. T2D and high ECV were both found to be independent risk factors for adverse CV outcomes.

## Introduction

Type 2 diabetes mellitus (T2D), which is a common chronic disease, is a well-recognized risk factor for heart failure (HF) independent of age, hypertension (HT), obesity, hypercholesterolemia, and coronary artery disease (CAD) (1). Patients with T2D have worse outcomes once HF has developed (2). The direct effect of hyperglycemia and insulin resistance on myocardial cellular metabolism may contribute to cardiac dysfunction by alteration of energy-substrate supply and impairment of metabolic-substrate switching under stress conditions. T2D also causes various morphologic changes of myocytes, extracellular matrix (ECM), and microvasculature. In addition, the accumulation of advanced glycation end products (AGEs) in the myocardium may contribute to HF events. AGEs increase both cardiac stiffening and collagen cross-linking in the myocardial ECM, both of which adversely affect systolic and diastolic cardiac function (3, 4). Since ECM expansion in humans is reversible, such as by inhibition of the renin-angiotensin-aldosterone system, quantification of ECM expansion may be a useful therapeutic marker for early cardiac involvement in patients with T2D.

Advanced cardiac magnetic resonance (CMR) imaging facilitates detailed, non-invasive characterization of the myocardium, including T1-mapping, and the derived parameter is extracellular volume fraction (ECV) (5). Some previous studies investigated the role of ECV in patients with diabetes and pre-diabetes compared to normal controls. Both of those studies reported increased ECV to be associated with a longer duration of diabetes, and that increased ECV may be associated with elevated glycated hemoglobin (HbA_1_c) level (6, 7). Another study found a significant association between diabetes and increased ECV, and that elevated ECV was significantly associated with an increased risk of adverse clinical outcomes, including HF and death (8).

This study aimed to investigate myocardial ECV by CMR T1 mapping compared between patients with and without T2D among patients with known or suspected CAD who were referred for CMR, and the effect of ECV and T2D on cardiovascular (CV) outcomes, including CV death, acute coronary syndrome (ACS), HF hospitalization, or ventricular tachycardia (VT)/ventricular fibrillation (VF).

## Methods

### Study Population

The study design was a retrospective cohort study. All patients aged >18 years with known or suspected CAD who underwent CMR for assessment of myocardial ischemia or myocardial viability at the Department of Cardiology of the Faculty of Medicine Siriraj Hospital, Mahidol University, Bangkok, Thailand during September 2017 to December 2018 were screened for inclusion eligibility. Native and post-contrast T1 maps were routinely performed in every clinical CMR study. All included patients were followed-up at our center for at least 6 months after the date of CMR. Patients diagnosed with cardiac amyloidosis or hypertrophic cardiomyopathy were excluded. Study patients were then allocated to either the T2D group or the non-T2D group. Patients included in the T2D group were either previously diagnosed as T2D at another center, had a documented diagnosis in their Siriraj Hospital medical record, were receiving anti-diabetic medications, or had a documented laboratory test of either fasting plasma glucose ≥126 mg/dl or HbA_1C_ ≥ 6.5% at least two times. The protocol for this study was approved by the Siriraj Institutional Review Board. Written informed consent was not obtained from study patients due to the retrospective confidentiality preserving nature of our study.

### CMR Image Acquisition

Cardiac magnetic resonance (CMR) was performed on an Ingenia 3.0T MR system (Phillips Healthcare, Best, The Netherlands) using ECG gating. The default CMR protocol includes a steady-state pre-precession sequence using the balanced-fast-field-echo technique of left ventricular (LV) short-axis, four-chamber, two-chamber, and three-chamber views, late gadolinium enhancement (LGE), and native and contrast-enhanced T1 mapping. LGE was performed by the three-dimensional segmented-gradient-echo inversion-recovery sequence.

T1 mapping was performed using modified Look-Locker inversion recovery (MOLLI) in a 5-(3)-3 scheme (5, 9). MOLLI was performed with breath-holding technique in mid-diastole in a single mid-ventricular short-axis slice (TR 2.2 ms, TE 1.8 ms, eight different TIs, matrix 152 × 150, field of view 300 × 300 mm^2^, flip angle 20°, SENSE 2, and 10-mm slice thickness).

### CMR Analysis

The basic analysis was performed using IntelliSpace Portal (ISP) software version 11.1 (Phillips Healthcare, Best, The Netherlands) by well-trained radiographers (10-year experience) and cardiologist fellows (3–5 year experience). Cine images were analyzed and LV volumetric data were obtained to derive left ventricular ejection fraction (LVEF). LGE images were analyzed by visual assessment based on the consensus of two readers and were interpreted as ischemic or non-ischemic (10). For ischemic LGE, the transmural extent of LGE was graded as a subendocardial or transmural scar for each myocardial segment according to the recommendation of the American Heart Association (AHA) (11). The analysis was blinded to the patient's name and functional images.

Native and contrast-enhanced T1 mapping was performed using CVI42 software version 5.12 (Circle Cardiovascular Imaging, Calgary, Alberta, Canada). The region of interest (ROI) was selected manually at the entire interventricular septum of the mid-cavity short-axis map while taking care to avoid imaging artifacts. According to the recommendation by the Society of Cardiovascular Magnetic Resonance (5), ROI for T1 mapping that was used to calculate ECV can be drawn at the septal segments or a complete single short-axis slice (usually a mid-ventricular slice). However, a single ROI drawn in the septum on mid-cavity short-axis maps is preferred to avoid lung, liver, and veins as sources of susceptibility artifacts. In another review article (12), the authors summarized that septal sampling has been shown to yield the greatest precision and minimize the effect of considerable variations of regional T1 values caused by the artifact-prone LV free wall myocardium. ECV was calculated using the following formula (13):


$$\text{ECV }\left(\%\right)\text{ = }(\text{1-Hematocrit})\;\times \;\left(\frac{\frac{\text{1}}{\text{Post contrast }{\text{T1}}_{\text{Myocardium }}}\text{-}\frac{\text{1}}{\text{Native }{\text{T1}}_{\text{Myocardium }}}}{\frac{\text{1}}{\text{Post contrast }{\text{T1}}_{\text{Blood }}}\text{-}\frac{\text{1}}{\text{Native }{\text{T1}}_{\text{Blood}}}}\right)\;\times \text{100}$$

According to a previous study that validated synthetic hematocrit (Hct) values derived from blood T1 obtained using a 3.0-T Philips MR system (Phillips Healthcare, Best, The Netherlands), we used the following formula to analyze the synthetic hematocrit (values between 0 and 1) for ECV (14):


$$\text{Synthetic Hct MOLLI = (869}{\text{.7 × [1/T1}}_{\text{blood}}\text{]) – 0.071}$$

### Data Collection

The following data were collected: age, gender, anthropometric data, underlying disease of HT, CAD, CKD, T2D, and the medications being used by the patient at the time of CMR. Collected laboratory data included hematocrit, serum creatinine, LDL, and HbA_1c_, and those data were collected as close to the date of CMR as possible.

### Outcome

The main outcome was a composite CV outcome consisting of CV death, ACS, HF hospitalization, or VT/VF. Data were collected from a medical record review. Events were collected from the time of CMR until the last follow-up visit by identifying the documented diagnosis of events by primary physicians and/or consulting cardiologists.

### Statistical Analysis

Continuous data are presented as mean plus/minus SD, and means between two groups were compared using the Student's *t*-test for unpaired data. Categorical data are presented as the number and percentage of patients, and differences between groups were analyzed using the chi-squared test. Baseline characteristics, laboratory data, and CMR data were compared between patients with and without the composite outcome. Univariate and multivariate analyses were performed to identify variables that predict the composite outcome. We used all data (demographic data, CV risk factors, laboratory data, and CMR data) except medications for the univariate model and then selected variables with a *p* < 0.05 from univariate analysis to run a multivariate model. Time-to-event analysis was performed using Cox regression, and the results are presented as Kaplan–Meier curves. The incremental prognostic value of variables in the final multivariate model was assessed using a Cox regression model based on clinical data, investigational data, and ECV values. The incremental value was assessed by considering these variables in hierarchical order, and by comparing the global chi-squared value derived from each hierarchical model. All analyses were performed using SPSS version 18 (SPSS, Inc., Chicago, IL, USA). A *p* < 0.05 indicates statistical significance.

## Results

**Figure 1:**
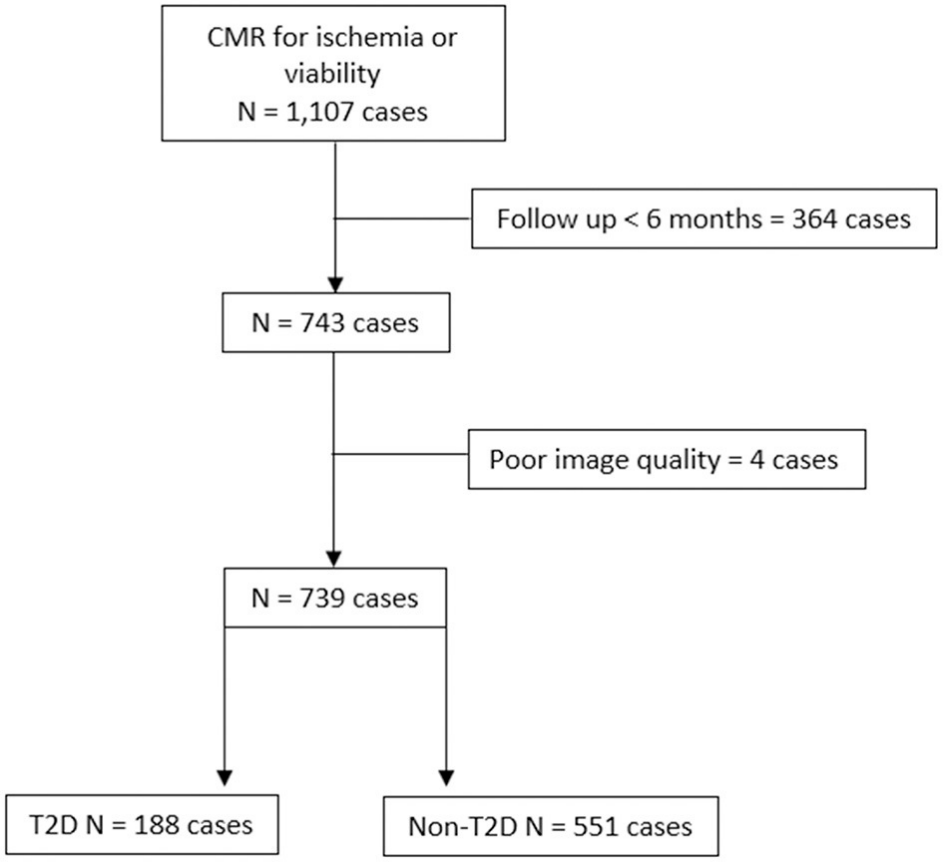
Flow diagram of the patient enrollment process.

During the study period, 1,217 subjects underwent contrast-enhanced CMR for assessment of myocardial ischemia or viability. After the exclusion of patients with unavailable follow-up data and/or poor image quality, 739 patients remained for the final analysis. Of those eligible patients, 188 (25.4%) were allocated to the T2D group. A flow diagram of the patient enrollment process is shown in Figure 1.

### Baseline Clinical and CMR Data of Patients With T2D and Non-T2D

Table 1 shows baseline patient characteristics compared between those with and without T2D. T2D subjects were significantly older (72.4 ± 10.5 vs. 68.5 ± 14.8 years, *p* = 0.024), had more comorbidities, and used more CV medications. In T2D group, the anti-diabetic medications used were metformin in 97 (51.6%), sulfonylurea in 78 (40.4%), thiazolidinediones in 21 (10.6%), DPP-4 inhibitors in 52 (27.7%), SGLT-2 inhibitors in 17 (9.0%), GLP-1 agonists in six (3.2%), and insulin in 14 (6.9%) patients. History of coronary disease confirmed by coronary angiogram before CMR was demonstrated in 92 patients (12.4%); 27 (29.4%), 28 (30.4%), and 37 (40.2%) had single, double, and triple vessel disease, respectively. Among patients with a coronary angiogram, 87 (94.6%) had coronary revascularization before CMR.

**Table 1:** Baseline demographic and clinical data of all patients, and compared between those with and without type 2 diabetes.

**Data**	**All** **(*N* = 739)**	**T2D** **(*n* = 188)**	**Non-T2D** **(*n* = 551)**	***p***
Age (years)	69.5 ± 14.0	72.4 ± 10.5	68.5 ± 14.8	***<0.001***
Male gender	364 (49.3%)	93 (49.5%)	271 (49.2%)	0.946
BMI (kg/m^2^)	25.4 ± 4.7	26.9 ± 5.6	24.8 ± 4.2	***<0.001***
Hypertension	418 (56.6%)	142 (75.5%)	276 (50.1%)	***<0.001***
Smoking	47 (6.4%)	17 (9.0%)	30 (5.4%)	0.081
Family history of CAD	7 (0.9%)	1 (0.5%)	6 (1.1%)	0.685
DLP	432 (58.5%)	143 (76.1%)	289 (52.5%)	***<0.001***
History of MI	57 (7.7%)	13 (6.9%)	44 (8.0%)	0.635
CKD	185 (25.0%)	88 (46.8%)	97 (17.6%)	***<0.001***
Cardiovascular medication:				
Beta-blockers	315 (42.6%)	93 (49.5%)	222 (40.3%)	***0.028***
CCB	216 (29.2%)	89 (47.3%)	127 (23.0%)	***<0.001***
Nitrates	138 (18.7%)	49 (26.1%)	89 (16.2%)	***0.003***
ACEI	83 (11.2%)	21 (11.2%)	62 (11.3%)	0.975
ARB	123 (16.6%)	39 (20.7%)	84 (15.2%)	0.080
Aldosterone antagonist	30 (4.1%)	5 (2.7%)	25 (4.5%)	0.260
Aspirin	317 (42.9%)	115 (61.2%)	202 (63.7%)	***<0.001***
P_2_Y_12_ inhibitors	114 (15.4%)	40 (21.3%)	74 (13.4%)	***0.010***
Statins	409 (55.3%)	152 (80.9%)	257 (46.6%)	***<0.001***
Laboratory data				
Hct (%)	38.8 ± 5.2	38.0 ± 5.2	39.2 ± 5.1	***0.010***
GFR (ml/min/1.73 m^2^)	56.2 ± 24.0	53.5 ± 24.5	60.0 ± 22.8	***0.018***
LDL (mg/dl)	83.7 ± 36.6	79.8 ± 38.8	89.2 ± 32.6	***0.026***
HbA1C (%)	6.2 ± 2.2	6.8 ± 2.0	5.1 ± 2.4	***<0.001***
CMR findings				
LVEF (%)	64.2 ± 17.8	64.7 ± 18.9	64.0 ± 17.4	0.599
LVEF <50%	134 (18.1%)	38 (20.2%)	96 (17.4%)	0.391
LGE present (%)	236 (31.9%)	73 (38.8%)	163 (29.6%)	***0.019***
T1 native (ms)	1,332 ± 63	1,335 ± 75	1,331 ± 58	0.516
ECV (%)	29.1 ± 5.0	30.0 ± 5.9	28.8 ± 4.7	***0.004***

*Data presented as number and percentage of patients or mean ± SD*.

*A p < 0.05 indicates statistical significance (bold-italic)*.

*T2D, type 2 diabetes; BMI, body mass index; CKD, chronic kidney disease; CAD, coronary artery disease; CCB, calcium channel blockers; ACEI, angiotensin converting enzyme inhibitors; ARB, angiotensin-receptor blockers; Hct, hematocrit; GFR, glomerular filtration rate; LDL, low-density lipoprotein-cholesterol; LVEF, left ventricular ejection fraction; ECV, extracellular volume fraction*.

Results of CMR demonstrated an average LVEF of 64.2 ± 17.8%. There were no significant differences in LVEF and native T1 between T2D and non-T2D; however, T2D had a greater proportion of LGE and significantly higher ECV compared to non-T2D. LV hypertrophy as defined by LV mass index more than 95% of healthy volunteers was demonstrated in 65 cases (8.8%). LGE was present in 236 patients (31.9%). The mean number of segments with the scar was 5.2 ± 3.7 (from the 16-segment model). Among those who had LGE, it was CAD pattern (subendocardial or transmural scar) in 78.8%, non-CAD pattern in 19.1%, and combined in 2.1%. Sixty-five out of 191 patients (34.0%) with CAD pattern LGE were asymptomatic. Subsequent management of patients with asymptomatic CAD was restricted to the adjustment of cardiac medications in 23 (35.4%) cases, whereas 15 (23.1%) underwent invasive angiography due to ischemia or other clinical indications. For those who underwent coronary angiography, 14 out of 15 (94%) had significant stenosis in at least one coronary artery. With patients exhibiting non-ischemic LGE, mid-wall scar, patchy scar, right ventricular insertion scar, and the subepicardial scar was detected in 18 (36%), 12 (24%), 10 (20%), and 18 (36%) cases, respectively. Eight patients had two patterns of nonischemic LGE.

Myocardial ischemia was observed in 229 (31.0%) cases. The mean number of ischemic segments was 3.4 ± 3.7. With patients exhibiting inducible ischemia, 104 (45.4%) had just adjustment of their cardiac medications, whereas 96 (41.9%) underwent invasive angiography. For those who had a coronary angiogram, significant stenosis of at least one major coronary artery was demonstrated in 91 (95.8%). Indirect evidence of diastolic dysfunction such as LV hypertrophy and left atrial enlargement was observed in 65 (8.8%) and 148 (20.0%) patients, respectively.

### CV Outcomes

The overall mean follow-up duration was 26.2 ± 8.5 months. Fifty-seven patients (7.7%) experienced death (*n* = 20), ACS (*n* = 7), HF hospitalization (*n* = 33), or VT/VF (*n* = 1) during follow-up. The composite outcomes (CV death, ACS, HF hospitalization, or VT/VF) occurred in 43 patients (5.8%). Comparisons of baseline clinical data and CMR data between patients with and without composite outcomes are shown in Table 2. Patients with a composite outcome had a higher proportion of T2D, CKD, CV drugs, LGE and lower Hct, GFR, and LVEF compared to those without the composite outcomes. Native T1 and ECV were higher in patients with composite outcomes.

**Table 2:** Baseline demographic and clinical data of all patients, and compared between those with and without cardiovascular composite outcome.

**Variables**	**Composite** **outcome** **(*n* = 43)**	**No composite** **outcome** **(*n* = 696)**	***p***
Age (years)	72.2 ± 14.1	69.3 ± 13.9	0.190
Male gender	24 (55.8%)	340 (48.9%)	0.375
BMI (kg/m^2^)	25.0 ± 5.0	25.4 ± 5.0	0.564
T2D	24 (55.8%)	164 (23.6%)	***<0.001***
Hypertension	25 (58.1%)	393 (56.5%)	0.830
Smoking	3 (7.0%)	44 (6.3%)	0.749
Family history of CAD	1 (2.3%)	6 (0.9%)	0.344
DLP	24 (55.8%)	408 (58.6%)	0.717
History of MI	8 (18.6%)	49 (7.0%)	***0.013***
CKD	21 (48.8%)	164 (23.6%)	***<0.001***
Laboratory data			
Hct (%)	37.2 ± 5.1	38.9 ± 5.2	***0.042***
GFR (ml/min/1.73 m^2^)	45.1 ± 24.7	57.5 ± 23.6	***0.006***
LDL (mg/dl)	83.8 ± 49.5	83.7 ± 34.8	0.991
HbA_1c_ (%) (*n* = 265)	6.8 ± 2.0	5.1 ± 2.4	***<0.001***
CMR findings			
LVEF (%)	49.1 ± 22.8	65.1 ± 17.0	***<0.001***
LGE present (%)	23 (53.5%)	213 (30.6%)	***0.002***
T1 native (ms)	1371 ± 93	1330 ± 60	***0.006***
ECV (%)	32.0 ± 7.0	29.0 ± 4.8	***0.008***

*Data presented as number and percentage of patients or mean ± SD*.

*A p < 0.05 indicates statistical significance (bold-italic)*.

*BMI, body mass index; T2D, type 2 diabetes; CKD, chronic kidney disease; CAD, coronary artery disease; CCB, calcium channel blockers; ACEI, angiotensin-converting enzyme inhibitors; ARB, angiotensin-receptor blockers; Hct, hematocrit; GFR, glomerular filtration rate; LDL, low-density lipoprotein-cholesterol; HbA_1C_, glycated hemoglobin; LVEF, left ventricular ejection fraction; ECV, extracellular volume fraction*.

### Univariate and Multivariate Analysis

**Figure 2:**
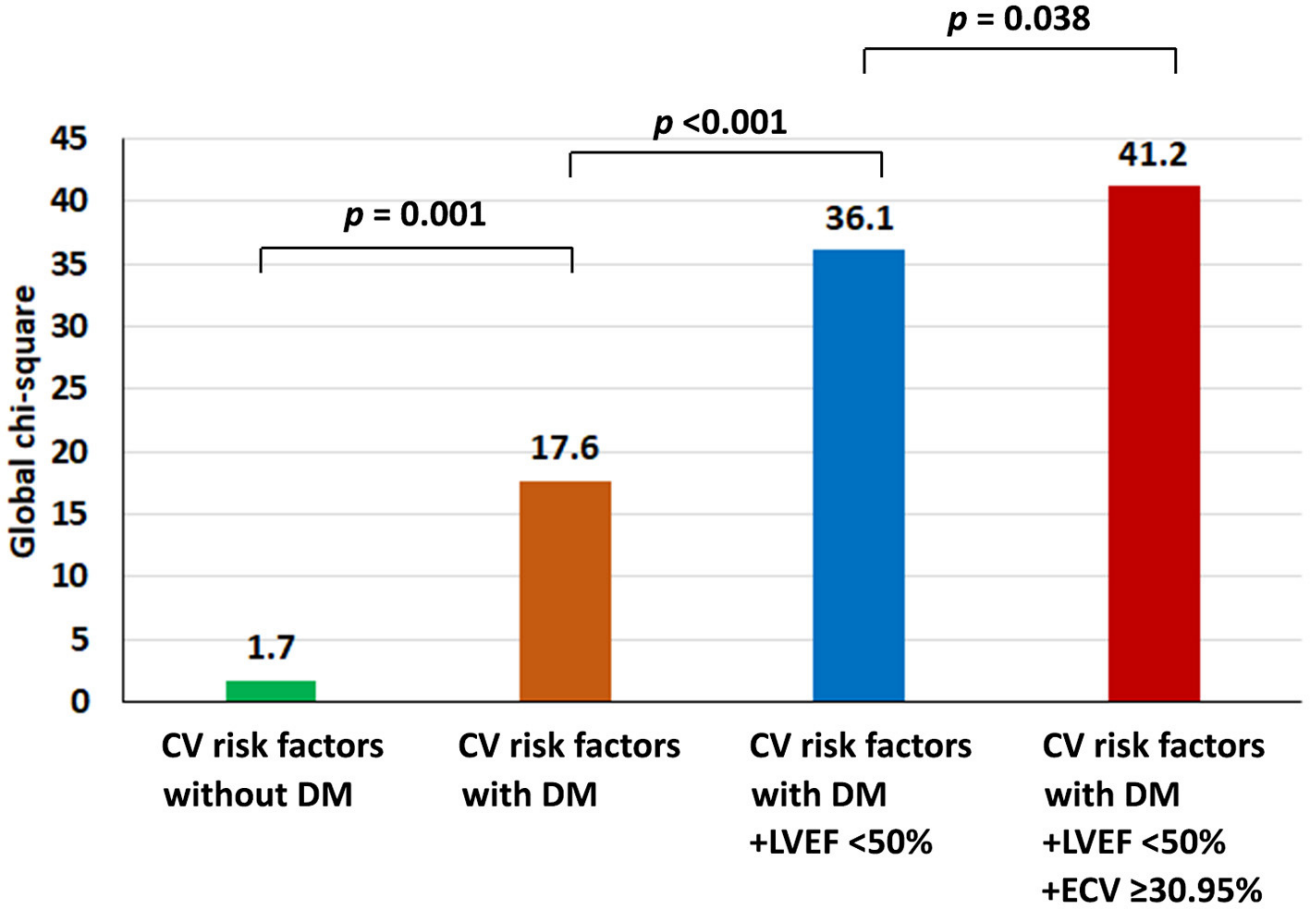
Incremental prognostic value shown as global chi-squared value compared among patients with type 2 diabetes (T2D); patients with T2D and left ventricular ejection fraction (LVEF) < 50%; and patients with T2D, LVEF < 50%, and extracellular volume fraction ≥30.95%.

All variables from Table 2 were used for both univariate and multivariate analyses to identify predictors of the composite outcome. ECV was classified as high when the ECV was in the top quartile. The cut-off for the top quartile of ECV was 30.95%. From multivariate analysis, T2D [hazard ratio (HR): 2.41, 95% CI: 1.17–4.98], high ECV (HR: 2.01, 95% CI: 1.03–3.93), and LVEF <50% (HR: 2.31, 95% CI: 1.10–4.88) were identified as independent predictors of CV events (Table 3). The addition of ECV data significantly improved the prognostic power of a model, namely, CV risk factors, T2D status, and LVEF data, with a significant increase in global chi-squared values from 1.7 with CV risk factors without T2D to 17.6 for CV risk factors with T2D, to 36.1 for CV risk factors with T2D and LVEF <50%, and to 41.2 for CV risk factors with T2D, LVEF < 50%, and ECV ≥ 30.95% (Figure 2).

**Table 3:** Univariate and multivariate analysis for independent predictors of cardiovascular composite outcome.

**Variables**	**Univariate analysis**		**Multivariate analysis**	
	**HR (95% CI)**	***P***	**aHR (95% CI)**	***p***
Age ≥ 65	1.22 (0.60–2.48)	0.583		
Male gender	1.32 (0.72–2.40)	0.373		
BMI ≥ 25	1.04 (0.57–1.90)	0.900		
T2D	2.78 (1.52–5.10)	***0.001***	2.76 (1.51–5.06)	***0.001***
Hypertension	1.07 (0.58–0.96)	0.830		
Smoking	1.08 (0.34–3.50)	0.894		
Family history of CAD	4.31 (0.59–31.49)	0.150		
DLP	0.83 (0.45–1.51)	0.530		
History of MI	3.10 (1.44–6.6.9)	***0.004***		
CKD	2.57 (1.41–4.69)	***0.002***		
GFR	2.72 (1.18–6.29)	***0.019***		
LVEF <50%	3.57 (1.93–6.59)	***<0.001***	3.11 (1.67–5.80)	***<0.001***
LGE present	2.58 (1.42–4.69)	***0.002***		
T1 native ≥ 1,367 ms	2.21 (1.17–4.16)	***0.014***		
ECV ≥ 30.95%	2.14 (1.14–4.04)	**0.019**	2.06 (1.12–3.79)	***0.020***

*A p < 0.05 indicates statistical significance (bold-italic)*.

*HR, hazard ratio; aHR, adjusted hazard ratio; T2D, type 2 diabetes; CKD, chronic kidney disease; ARB, angiotensin-receptor blockers; GFR, glomerular filtration rate; LVEF, left ventricular ejection fraction; ECV, extracellular volume fraction*.

### Survival Analysis

**Figure 3:**
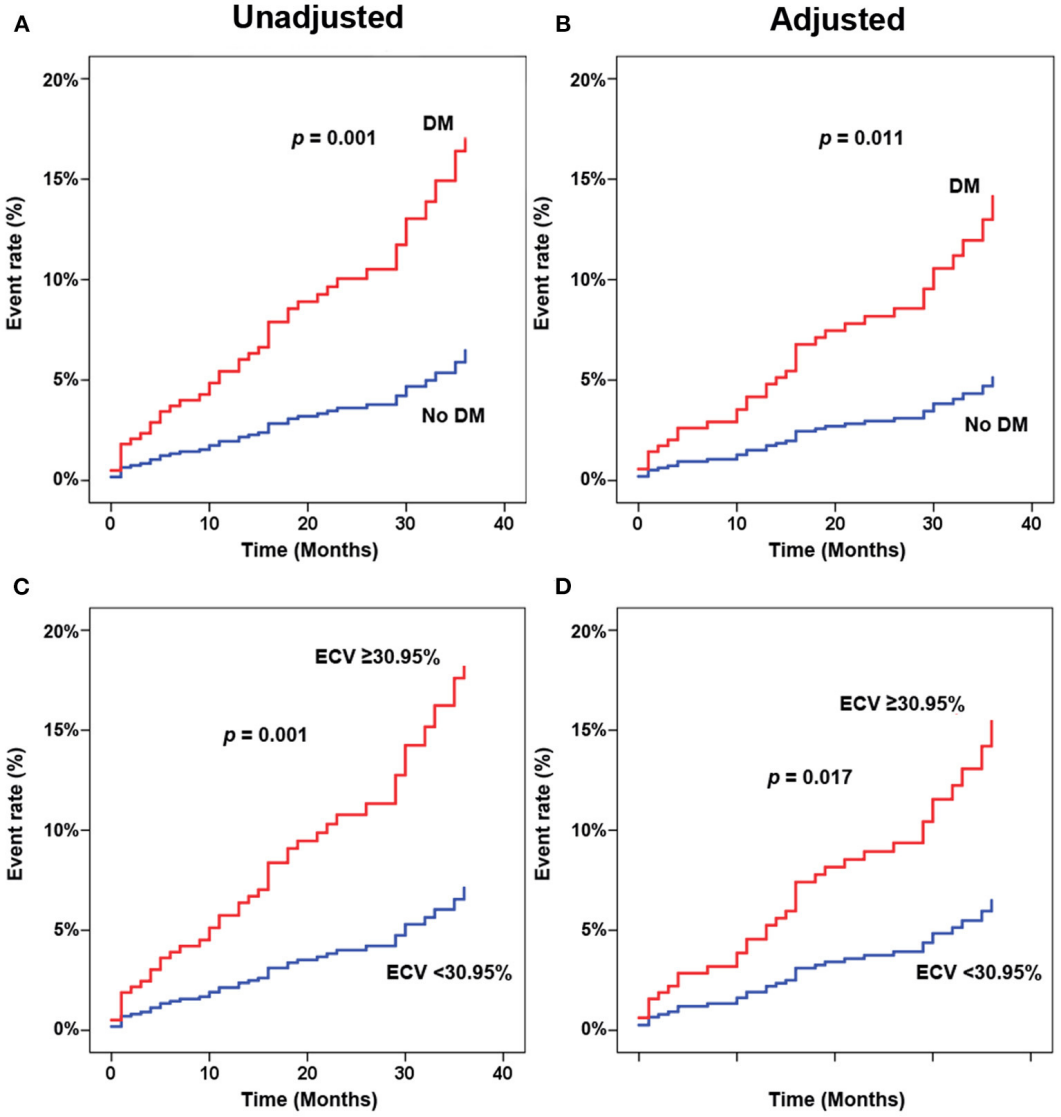
Adjusted **(A,C)** and unadjusted **(B,D)** hazard graphs of the cumulative event rate compared between patients with and without type 2 diabetes (T2D) **(A,B)**, and between patients with extracellular volume fraction (ECV) above and below the cut-off value.

Figure 3 shows adjusted and unadjusted hazard graphs of the cumulative event rate compared between patients with and without T2D, and between patients with ECV above and below the 30.95% cut-off value. Patients with T2D and patients with high ECV both had an increased incidence of CV composite outcomes over time.

**Figure 4:**
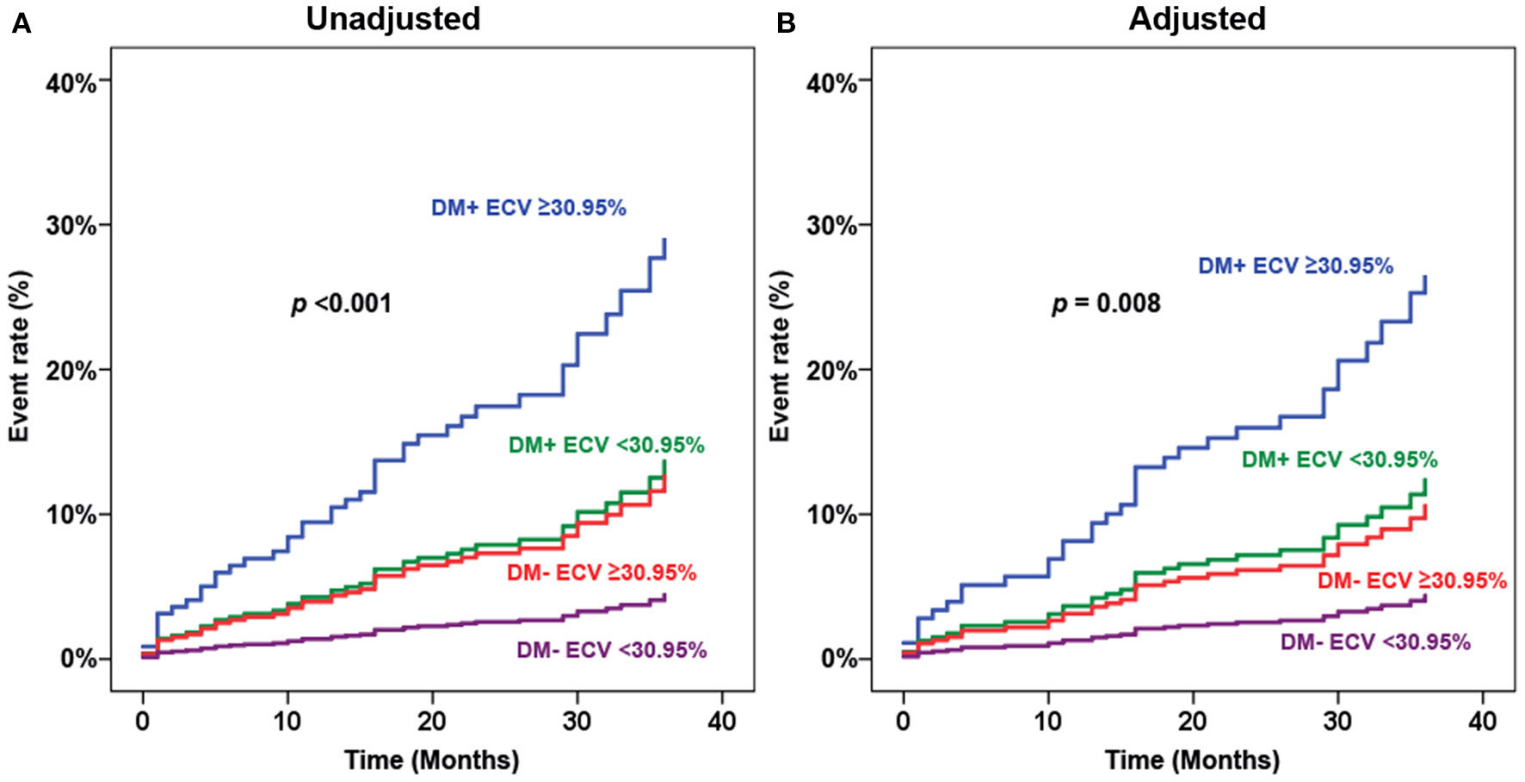
Adjusted **(A)** and unadjusted **(B)** hazard graphs of the cumulative event rate compared among four groups, namely, (1) type 2 diabetes (T2D) and extracellular volume fraction (ECV) ≥ 30.95%, (2) T2D and ECV < 30.95%, (3) non-T2D and ECV ≥ 30.95%, and (4) non-T2D and ECV < 30.95%.

**Figure 5:**
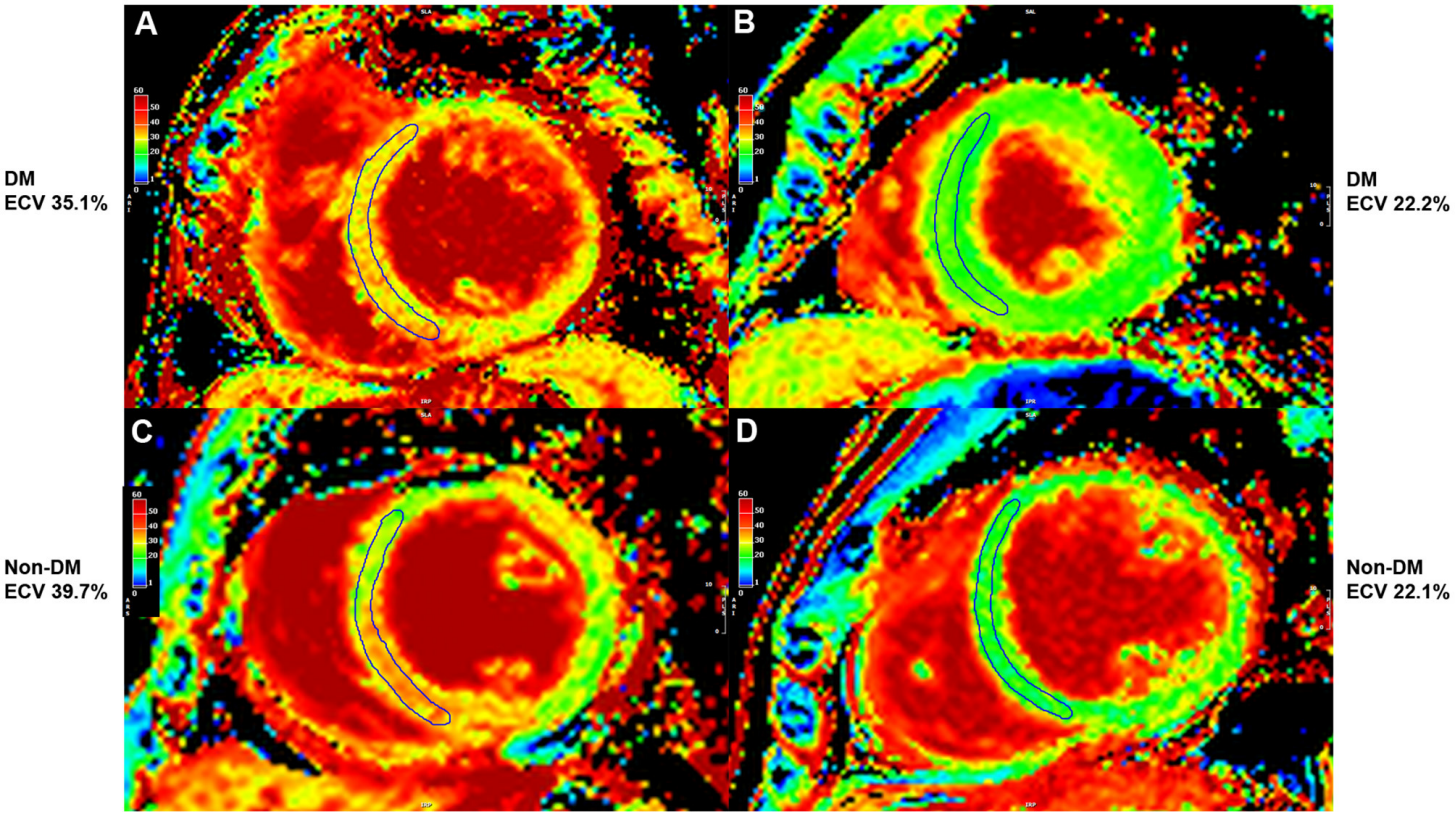
Extracellular volume fraction (ECV) mapping of patients with type 2 diabetes (T2D) and high ECV **(A)**, T2D and lower ECV **(B)**, high ECV without T2D **(C)**, and lower ECV without T2D **(D)**.

Figure 4 shows adjusted and unadjusted hazard graphs of the cumulative event rate compared among four groups, namely, (1) T2D and ECV ≥ 30.95%, (2) T2D and ECV < 30.95%, (3) non-T2D and ECV ≥ 30.95%, and (4) non-T2D and ECV < 30.95%. Both graphs demonstrate that the highest event rate was among patients with T2D and high ECV, and the lowest event rate was among patients with non-T2D and lower ECV. Patients with only one of these two factors had a prognosis in between the two aforementioned groups. Figure 5 shows ECV mapping of patients with T2D and high ECV, T2D and lower ECV, high ECV without T2D, and lower ECV without T2D.

### Sensitivity Analysis for ECV Data

**Figure 6:**
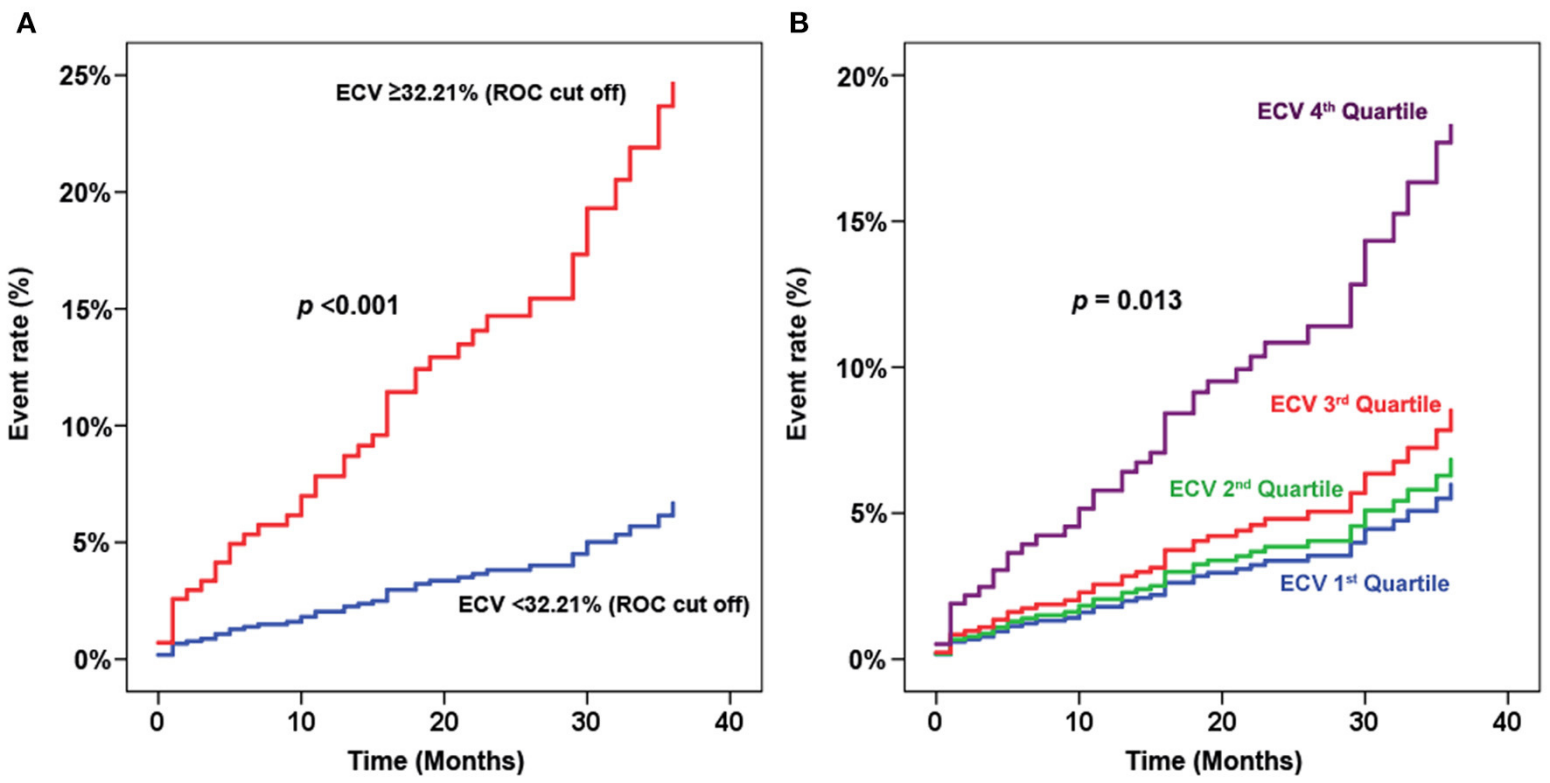
Sensitivity analysis of extracellular volume fraction (ECV) for predicting clinical outcomes. **(A)** Hazard graph of patients with ECV above and below the cut-off value derived from receiver operating characteristic (ROC) curve analysis. **(B)** Hazard graph of each quartile of ECV.

To explore whether the significant finding of the predictive value of ECV remains significant when comparing ECV by methods other than the fourth quartile compared to the other three quartiles, we performed a sensitivity analysis of ECV for predicting clinical outcomes, including a hazard graph of patients with ECV above and below the cut-off value derived from receiver operating characteristic curve analysis (Figure 6A), and a hazard graph of each quartile of ECV (Figure 6B). Both of those sensitivity analyses showed a higher ECV to be significantly associated with an increased risk of adverse clinical outcomes. This finding reflects the predictive ability of ECV in this clinical setting.

We also performed a sensitivity analysis using ECV based on the results of actual Hct with 6 months before CMR which was available in 358 (48.4%) patients. ECV derived from the actual Hct had an HR and 95% CI for clinical outcomes of 3.37 (1.62–6.98). Multivariate analysis using ECV derived from actual Hct demonstrated that T2D, LVEF < 50%, high ECV (the fourth quartile of ECV and history of myocardial infarction were in the final model. The HR and 95% CI of ECV in the multivariate model were 2.50 (1.18–5.28).

## Discussion

This retrospective cohort study focused on the relationship between T2D and ECV, and the influence of these two factors on the clinical outcomes of patients who were referred for CMR for assessment of myocardial ischemia or viability. Our results showed ECV to be significantly higher in T2D than in non-T2D. Regarding CV outcome, T2D and high ECV were both found to be independent predictors of composite CV outcome. Patients with coexisting T2D and high ECV were shown to be at significantly higher risk of experiencing an adverse CV outcome.

T2D was previously reported to be significantly associated with an increased risk of CV outcomes, namely, HF (1), sudden cardiac death (15), and myocardial infarction (16). Our results also showed T2D to be significantly associated with composite CV outcome (HR: 2.95, 95% CI: 1.24–7.01), which is consistent with the results of previous studies.

A previous study reported that T2D was associated with higher ECV compared to non-T2D [30.2%, interquartile range (IQR): 26.9–32.7% vs. 28.1%, IQR: 25.9–31.0, respectively; *p* < 0.001] (8), and that higher ECV was significantly associated with the combined endpoints of death or incident HF admission for both patients with T2D (HR: 1.52, 95% CI: 1.21–1.89) and patients with non-T2D (HR: 1.46, 95% CI: 1.25–1.71). Our study showed that patients with T2D had higher ECV. T2D, high ECV, and LVEF < 50% were all found to be independent predictors of an increased risk for adverse CV outcomes. The impact of T2D and high ECV on CV outcomes was independent of LVEF. ECV was associated with worse composite CV outcomes (HR: 2.01, 95% CI: 1.03–3.93). We also showed the incremental prognostic value of the factors that independently predict composite outcomes that were derived from the final multivariate model, including T2D, LVEF < 50%, and high ECV. In addition, we demonstrated that patients with coexisting T2D and high ECV were associated with a higher risk of adverse CV outcomes.

Although LGE was a significant predictor for clinical outcome in the univariate analysis, it was removed from the final multivariate model. LGE, LVEF < 50%, and high ECV are variables derived from CMR and the three variables may have different impacts on the incremental prognostic value. We tested this hypothesis by running an analysis on the incremental prognostic value three times with the simulation of the presence of the data on two components and adding the third component. We found that with the presence of data of LVEF < 50%, and high ECV, adding LGE data did not significantly increase the prognostic value. However, LVEF < 50% or high ECV significantly increase the prognostic value when they were added as the third variable. This finding means that LVEF < 50% and high ECV were more significant predictors than LGE.

Late gadolinium enhancement (LGE) is a good predictor of clinical outcomes in patients with T2D (17). Stress CMR images had an add-on predictive value on top of LGE in patients with and without T2D (18, 19). It helps reclassify risk in patients with T2D who were referred for stress CMR. LGE is a hallmark for poor outcomes in patients with T2D whereas myocardial ischemia was a good predictor both in patients with and without T2D (19). Our study explores the predictive value of ECV which is another aspect of CMR in patients with T2D.

As mentioned earlier, T2D is associated with various morphologic changes to myocytes, ECM, and microvasculature, which individually and in combination exert an adverse influence on CV outcomes. T2D effectuates myocardial ECM expansion *via* the accumulation of AGEs, with reported resulting myocardial fibrosis, systolic and diastolic dysfunction (20), vasomotor dysfunction (21), arrhythmia (22), and mortality (8). The results of this study prove that patients with T2D have higher ECV, which has a strong negative impact on worse CV outcomes when compared to patients with non-T2D.

We demonstrated that high ECV is an independent predictor for the adverse CV outcomes in patients with T2D. High ECV added prognostic value on top of CV risk factors, T2D status, and low LVEF. Although LGE data is an independent prognostic factor in patients with T2D (17), it was not an independent predictor in the presence of T2D, low LVEF, and ECV data.

### Limitations

This study has some limitations. First, our study had a retrospective design. Given that our data were retrospectively collected and hematocrit level was not routinely required before CMR, we did not have available hematocrit data for all patients. To compensate, we used a synthetic hematocrit formula to derive ECV in this study. Second, the data included in this study was from a single center. Third, this is a retrospective cohort study based on the existing CMR data to answer the research question. Therefore, we did not have healthy normal as a control group. However, our study aimed to determine, among patients with known or suspected CAD who were referred for CMR, the difference in ECV between T2D and non-T2D and to determine the influence of T2D and ECV on composite clinical outcome. A multicenter study in a much larger study population and longer follow-up duration may yield greater insight into the relationship between T2D and ECV, and may identify additional risk factors for adverse CV outcomes. Furthermore, the effect of medication changes on CMR variables, including ECV merits further investigation in future studies.

## Conclusion

Among patients with known or suspected CAD, ECV was higher in patients with T2D than in patients with non-T2D. T2D, low LVEF, and high ECV were both identified as independent predictors of adverse CV outcomes. Patients with T2D with coexisting high ECV were strongly associated with adverse CV outcomes.

## Data Availability Statement

The original contributions presented in the study are included in the article/supplementary material, further inquiries can be directed to the corresponding author.

## Ethics Statement

The protocol for this study was approved by the Siriraj Institutional Review Board. The Ethics Committee waived the requirement of written informed consent for participation.

## Author Contributions

Concept and design, data acquisition, interpretation of data, manuscript preparation, manuscript revision, and manuscript review by IL and RK. Data acquisition, data analysis, manuscript revision, and manuscript review by TS, YK, and AY. All authors read, approved the final manuscript, and approved the submission of this manuscript for journal publication.

## Conflict of Interest

The authors declare that the research was conducted in the absence of any commercial or financial relationships that could be construed as a potential conflict of interest.

## Publisher's Note

All claims expressed in this article are solely those of the authors and do not necessarily represent those of their affiliated organizations, or those of the publisher, the editors and the reviewers. Any product that may be evaluated in this article, or claim that may be made by its manufacturer, is not guaranteed or endorsed by the publisher.
